# Activity-based anorexia animal model: a review of the main neurobiological findings

**DOI:** 10.1186/s40337-021-00481-x

**Published:** 2021-10-02

**Authors:** Sara Spadini, Mattia Ferro, Jacopo Lamanna, Antonio Malgaroli

**Affiliations:** 1 Center for Behavioral Neuroscience and Communication (BNC), Vita‐Salute San Raffaele University, Via Olgettina 58, 20132 Milan, Italy; 2 Department of Psychology, Sigmund Freud University, Milan, Italy; 3 Faculty of Psychology, Vita-Salute San Raffaele University, Milan, Italy

**Keywords:** Anorexia nervosa, Activity-based anorexia, ABA, Animal model, Eating disorders, Behavior, Psychiatric disorder

## Abstract

**Background:**

The genesis of anorexia nervosa (AN), a severe eating disorder with a pervasive effect on many brain functions such as attention, emotions, reward processing, cognition and motor control, has not yet been understood. Since our current knowledge of the genetic aspects of AN is limited, we are left with a large and diversified number of biological, psychological and environmental risk factors, called into question as potential triggers of this chronic condition with a high relapse rate. One of the most valid and used animal models for AN is the activity-based anorexia (ABA), which recapitulates important features of the human condition. This model is generated from naïve rodents by a self-motivated caloric restriction, where a fixed schedule food delivery induces spontaneous increased physical activity.

**Aim:**

In this review, we sought to provide a summary of the experimental research conducted using the ABA model in the pursuit of potential neurobiological mechanism(s) underlying AN.

**Method:**

The experimental work presented here includes evidence for neuroanatomical and neurophysiological changes in several brain regions as well as for the dysregulation of specific neurochemical synaptic and neurohormonal pathways.

**Results:**

The most likely hypothesis for the mechanism behind the development of the ABA phenotype relates to an imbalance of the neural circuitry that mediates reward processing. Evidence collected here suggests that ABA animals show a large set of alterations, involving regions whose functions extend way beyond the control of reward mechanisms and eating habits. Hence, we cannot exclude a primary role of these alterations from a mechanistic theory of ABA induction.

**Conclusions:**

These findings are not sufficient to solve such a major enigma in neuroscience, still they could be used to design ad hoc further experimental investigation. The prospect is that, since treatment of AN is still challenging, the ABA model could be more effectively used to shed light on the complex AN neurobiological framework, thus supporting the future development of therapeutic strategies but also the identification of biomarkers and diagnostic tools.

**Plain English summary:**

Anorexia Nervosa (AN) is a severe eating disorder with a dramatic effect on many functions of our brain, such as attention, emotions, cognition and motion control. Since our current knowledge of the genetic aspects behind the development of AN is still limited, many biological, psychological and environmental factors must be taken into account as potential triggers of this condition. One of the most valid animal models for studying AN is the activity-based anorexia (ABA). In this model, rodents spontaneously limit food intake and start performing increased physical activity on a running wheel, a result of the imposition of a fixed time schedule for food delivery. In this review, we provide a detailed summary of the experimental research conducted using the ABA model, which includes extended evidence for changes in the anatomy and function of the brain of ABA rodents. The hope is that such integrated view will support the design of future experiments that will shed light on the complex brain mechanisms behind AN. Such advanced knowledge is crucial to find new, effective strategies for both the early diagnosis of AN and for its treatment.

## Background

Anorexia nervosa (AN) is a complex eating disorder characterized by refusal of food and high fear of gaining weight. According to DSM-5 criteria, incidence is around 1% with a mortality rate of 5–10% [1]. AN primarily affects females (90%) and has the most frequent onset in adolescence, between 14 and 18 years of age [2]. Excessive physical activity is commonly observed in patients with AN [3] and correlates with poor clinical outcome [4], longer periods of hospitalization [5], high rates of relapse after recovery [6] and increased psychopathology [7].

Several studies explored the neurobiological mechanisms of this condition by identifying molecules, brain circuits and genetic characteristics of patients [8, 9]. Nevertheless, a clear picture about the etiopathology of AN, as for other important eating disorders, is still missing, and this heavily limits the development of new, more effective treatments. Valid animal models of AN proved to be particularly difficult to develop due to the spontaneous nature of caloric restriction which is the main behavioral feature of the disease [10]. However, a self-starvation model based on excessive physical activity and temporal restriction of food availability showed high validity several decades ago and was named activity-based anorexia (ABA). The ABA model, in its many procedural variants, has been extensively applied to gather insights into neurochemical, neuroanatomical and behavioral changes induced by—or maybe driving—fasting and weight loss in rodents. Here, we review the main findings obtained using this model to provide an updated picture to support both the formulation of new hypotheses on the etiopathology of AN and the ideation of future experiments.

## Results

### Activity-based anorexia (ABA) model

In 1925, John F. Dashiell reported that rats, when deprived of water or food, increased their seeking behavior. This was interpreted as adaptive, since in wild life the exploration activity increases chances of finding nourishment. Some years later, Hall and Hanford showed that the level of physical activity, measured with the use of a running wheel, was significantly higher in animals subjected to a restricted feeding schedule compared to those allowed to an unrestricted access to food [11]. Starting from these observations, Routtenberg and Kuznesof studied in detail the behavior of rats initially kept under unlimited access to a running wheel [12]. Their running wheel activity (RWA) increased during the first two weeks and then stabilized, together with an increased intake of food, available ad libitum, a likely homeostatic compensation for increased energy expenditure. The same animals were then subjected to a food restriction program for 5 consecutive days, while water was always present. Food supply was limited to a single daily session of 1–2 h at a fixed day time, generally at the beginning of the dark phase, when crepuscular animals, including rats and mice, usually feed, and this caused a strong preference for RWA despite the chance of nourishment. Such behavior did not occur when animals were fed ad libitum and had constant access to a running wheel, nor in animals subjected to food restriction without a running wheel. Interestingly, food intake significantly increased if multiple daily feeding sessions were regularly set, *i.e.* if the access to food was changed from 1 h per day to 30 min twice a day, or 15 min four times a day. In contrast, irregular feeding resulted in lower food intake and increased RWA [13]. Nevertheless, once animals lose 20–25% of their weight, investigators intervene to prevent animals’ death by a recovery program consisting in blocking access to the wheel and restoring ad libitum access to food [14].

These behavioral outcomes are indeed the defining features of the ABA phenotype, the mostly used animal model for AN: increased physical activity, self-motivated restriction of food intake and substantial weight loss. Likewise, the plausible self-motivated restriction could be associated to exercise in the stabilization of food intake, suggesting a functional role of activity in this behavior [15]. In these animals, RWA is increased especially during the dark phase and starts 2–3 h before food presentation [16]. Such food anticipatory activity (FAA) closely relates to behaviors associated with reward seeking and anticipation of palatable stimuli [17], even if its ability to be used as a predictor of severity is under debate [18]. In addiction, RWA can be more easily triggered in female rodents and during animals adolescence [19–21]. Anhedonia, a trait present in patients with AN, is thought to emphasize weight loss in the ABA model as well [22]. Other typical alterations shown by ABA animals are hypothermia [23], amenorrhea [24], hypoleptinemia [25], ulcers [21, 26], increased activity of the hypothalamic–pituitary–adrenal (HPA) axis [27], impairment in cognitive functions such as learning and memory [28–33] and cognitive flexibility [34], disruption of gut microbiota, proteolysis and fatty acids breakdown as a consequence of food restriction [35, 36].

### The “suppression” and “mal-adaptation” theories

The underlying causes of ABA phenotype development are still unknown and speculative. According to the “suppression theory” [37], RWA is strengthened by food deprivation because motor activity replaces food as a primary reward. This hypothesis is strengthen by studies where it has been found that physical activity triggers addiction via the release of endogenous opioids and endorphins affecting dopamine reward circuits [38, 39]. A different “*mal-*adaptation theory” posits that physical exercise perturbs the adaptation to food restriction once the anticipatory behavior has developed, *i.e.* ABA arises because of an inability to cope with the new feeding schedule [40]. Actually, food restriction preceding running wheel presentation enhances dysfunctional behaviors as those induced by the ABA protocol [40, 41], although such enhancement has not been observed in all studies using the same pre-exposure protocols [42]. Additional theories have been put forward and could be integrated with the above: (1) food restriction induces foraging and migration behaviors considered to be adaptive and self-sustaining in order to cope with food shortage [43]; (2) since cold can trigger RWA, a behavioral loop aimed at increasing thermogenesis has been hypothesized, driven by a homeostatic response to hypothermia [14].

### Morphological and functional changes

#### Hippocampus and cerebral cortex

Anatomical changes have been mostly investigated in the hippocampus, the brain structure involved in memory, spatial and cognitive functions, and in anxiety regulation [44, 45]. Modifications in the hippocampus are largely influenced by hormonal changes that take place during adolescence [46] and have been found in AN patients suffering from anxiety and stress symptoms [47–49].

In female rats, after the induction of the ABA phenotype, the mean brain volumes were found to be reduced when compared to controls, as well as gliogenesis and number of astrocytes, analyzed in different brain structures such as cerebral cortex, dentate gyrus, dorsal hippocampus and corpus callosum [19, 50]. In particular, the cerebral cortex and the corpus callosum resulted significantly reduced in volume in the ABA phenotype, mirroring volumetric brain changes found in human subjects with AN [51]. This could affect metabolic and homeostatic maintenance of neurons and has been also associated with depression, which is in turn often linked to AN [52]. Neuronal plasticity changes induced by the ABA protocol, as well as by food restriction or RWA alone, have been investigated on apical ventral hippocampal dendrites of CA1 (involved in the regulation of stress) and dorsal hippocampus (involved in spatial memory): reduced dendritic length and decreased branching were observed in the dorsal hippocampus of ABA rats, compared to controls. Dendritic branching was instead increased in the *stratum radiatum* of the ventral hippocampus [53]. Such dendritic hypertrophy may be linked to enhanced expression of brain-derived neurotrophic factor (BDNF) driven by physical exercise [54]. As the matter of fact, BDNF was already shown to promote dendritic growth and branching at hippocampal pyramidal cells [55]. Long-term effects on hippocampal plasticity were also investigated in adolescent female rats prior to ABA induction, during it and after ABA relapse: decreased branching and early dendritic pruning were observed after ABA relapse, suggesting an earlier closure of the critical period and an age dependence for morphological changes in response to stress and anxiety, since such modifications were absent in adult ABA-induced animals [20]. Moreover, a reduced GABA innervation in CA1 pyramidal cells was analyzed by electron microscopy in susceptible animals, hypothesizing that low GABAergic levels and consequent excitability may explain food restriction-induced hyperactivity [56]. Importantly, activation of HPA by stress and high levels of corticosteroids, as those found in ABA animals [57], are known to affect synaptic transmission and plasticity both in the hippocampus [57, 58] and prefrontal cortex (PFC) [59–61]. Thus, mal-adaptive stress response might underly those synaptic plasticity changes observed in ABA animals [62]. Interestingly, loss in body weight and PFC metabolism seem to be positively correlated in ABA rats [63], while the length of GABAergic, soma-targeting GAD + terminals in the medial portion of the same region (mPFC) was found ~ 40% longer in ABA mice [64].

#### Hypothalamus

Hypothalamus is involved in neurohormonal regulation of the feeding behavior and in energy homeostasis [65]. Hypothalamic proteome, more specifically those proteins involved in mitochondrial metabolism, resulted modified in ABA mice, together with an increased mitochondrial fission [66]. Since electrical stimulation has claimed clinical benefits in patients affected by obsessive–compulsive disorder and major depression [67], different protocols of hypothalamus stimulation were tested in ABA animals before food access, but no changes in either RWA or food intake were recorded. Interestingly, when this region was electrically stimulated prior to the induction of the ABA phenotype, a decrease in RWA was noticed [68]. The dorsomedial hypothalamus, a region known for signaling promotion of feeding behavior [69], showed an increased neural activity in the ABA state [70]. A similar result was detected in other regions: in the hypothalamic suprachiasmatic and arcuate nuclei, in the locus coeruleus (which receives information from the dorso-medial hypothalamus), in the solitary tract nucleus as well as in other nuclei involved in regulation of food intake, physical activity, thermoregulation, depression, stress and anxiety [71].

#### Cerebellum

An increased metabolism was found in the cerebellum of ABA rats, when compared to controls, while hippocampus, striatum and thalamus showed reduced signals [72]. A separate study also found increased glucose metabolism in cerebellum, ventral pontine nuclei and dorsal thalamus of ABA rats [63]. Allowing rats to access a running wheel, with or without food restriction, revealed an increased density of noradrenergic fibers in the cerebellar vermis, that receives inputs from the spinal cord and the motor cortex for balance and movement control. On the contrary, in advanced ABA stages, excessive exercise was found to decrease noradrenergic fiber density [73].

#### Nucleus accumbens and striatum

The nucleus accumbens (NAc), the ventral portion of the striatum sending afferents to the ventral pallidum, the substantia nigra and the pontine reticular formation, is part of the reward system and was found to have an important role in FAA [74, 75]. This nucleus receives extended dopaminergic (DA) afferents from the ventral tegmental area (VTA), involved in the processing of food rewards and motivation to eat, as well as serotoninergic (5-HT) projections from the dorsal raphe. However, neither DA nor 5-HT were found to be increased in the NAc of ABA rats when compared to controls, while DA was found to be increased during normal feeding. Consequently, the hyperactivity in the early stage of FAA could not be explained with an increase of DA in these structures [76]. However, experimental rats showed an increased expression of GluA1 AMPA (α-amino-3-hydroxy-5-methyl-4-isoxazolepropionic acid) receptor subunits in NAc during the ABA acute phase, suggesting a role for glutamatergic transmission changes in the anomalous processing of reward observed in AN [77].

#### Gastrointestinal system

A possible association of intestinal abnormalities with AN pathophysiology has been investigated. One study found that the colon of ABA mice, if compared to control animals subjected to standard food restriction, showed reduced thickness of the muscular layer and increased mucosal permeability associated with lower expression of the tight junction proteins claudin-1 and occluding [78]. Another study on ABA mice found higher colon permeability associated with an increased ratio between lean mass and fat mass, and increased fat oxidation: a higher mucosal permeability persisted in mice that were re-fed with no access to the wheel, while a moderate physical activity during the re-feeding phase reduced colonic hyperpermeability without altering gastro-intestinal mucosa protein synthesis [79].

### Neurotransmission systems

Several pharmacological studies investigated the role of dopaminergic, serotoninergic and GABAergic signaling in the ABA model, based on the known dysregulation of such neurotransmitter systems found in AN, as well as on the ameliorating action of drugs acting on the associated receptors. As the matter of fact, in patients with AN, dopaminergic and serotoninergic dysfunctions have been extensively reported [9, 80–82] and drugs acting on receptors for these neurotransmitters have been widely used as treatments [83]. One of the most recent, olanzapine, is reported to act promiscuously as dopamine antagonist (D1-D5 receptors), serotonergic antagonist (5-HT 1A, 5-HT 3, 5-HT 6, 5-HT 7), inverse agonist (5-HT 2A, 5-HT 2B, 5-HT 2C receptors), adrenergic and muscarinic antagonist [84].

#### Dopamine and serotonin

One-week chronic treatment with olanzapine prior ABA induction reduced the phenotype in terms of RWA, starvation-related hypothermia and endocrine abnormalities in the HPA axis. Similar results were also found in control rats, fed ad libitum, suggesting a sedative effect leading to the reduction of the RWA [85]. A different study evidenced an increased survival and a reduced FAA in two mouse strains (Balb/6J and A/J) sub-chronically and chronically treated with olanzapine, compared to the selective serotonin reuptake inhibitor (SSR) fluoxetine [86]. The authors suggest that olanzapine might act either by modulating HPA axis response or by reducing the activity of the reward system, which seems altered in the ABA phenotype [37], thus recovering from abnormalities in dopaminergic signaling. In fact, physical activity activates dopaminergic pathways underlying reward similarly to addictive substances, thus forcing animals to undertake an intense physical exercise loop leading to extreme starvation, an effect possibly opposed by olanzapine thanks to its blocking action on one or more dopaminergic receptors [87]. Interestingly, other researchers suggested that the A/J mouse strain exploited above should be further used to investigate chromosomal regions related to excessive physical activity, since such behavior might represent a compensatory reward for food absence, hypothesis that is very close to the already described Pierce’s suppression theory [88].

Various studies have shown an alteration of dopamine receptor binding in eating disorders: a decreased availability of dopamine D2 receptor in the striatum of obese subjects [89] was reported, together with alterations in dopamine receptors expression in the striatum of AN patients [82] and an increased D2 receptor expression in the striatum of ABA mice [90]. Experimental overexpression of D2 receptors in the NAc of mice induced an altered glucose metabolism, driving weight loss only in females after the ABA protocol has started [91]. The use of D2/D3 receptor antagonists (eticlopride, amisulpride, L-741.626 and SB 27701A) actually reduced weight loss in ABA mice, if compared to controls, with a consequent increase in survival [92]. Finally, a mice strain knockout for the Na^+^/Cl^−^ dependent dopamine transporter (DAT) showed no hyperactivity associated to food restriction [88].

As previously mentioned, also 5-HT dysfunctions are involved in AN and SSRIs are used in the treatment of its symptoms, although their effects in terms of food intake and regulation of excessive physical activity are under debate [93, 94]. The ABA protocol has been shown to increase 5-HT at the hypothalamic level. This effect was canceled by tyrosine administration, improving food consumption as well as cognitive functions [95, 96]. Similarly, the SSRI fluvoxamine, if administered simultaneously to ABA induction, decreased RWA with no substantial increase in body weight [97]. Carbohydrates and tryptophan-containing food are known to increase 5-HT levels; hence, in patients suffering from AN, the reduced levels of these macronutrients cause low serotonin levels which in turn reduce anxiety and dysphoric behavior [9]. Consistently, the administration of 3,4-methylenedyoximethamphetamine (MDMA), a substance known to increase both dopamine release and activity of the postsynaptic 5-HT receptors, was shown to increase RWA and weight loss in rats subjected to ABA [98, 99]. The involvement of 5-HT in the etiology of AN seems also confirmed by the use of 8-OH-DPAT, a selective activator of 5-HT1A autoreceptors, which acts by diminishing serotonergic tone. When this drug was administered to ABA animals it reduced weight loss likely by limiting RWA [100]. At the same time, chronic administration of D-fenfluoramine, an indirect agonist of 5-HT that blocks its reuptake but also increases its release, accelerated weight loss in animals subjected to ABA protocol [101]. However, a different study using the same drug did not show a decrease of food intake in the ABA models nor an increase in exercise, but appeared to decrease water consumption, increasing the plasmatic osmolarity and the expression levels of arginine-vasopressin in the hypothalamus [102]. Lastly, rats subjected to ABA protocol were treated with chronic doses of agmatine, an arginine derivative which acts on multiple targets by blocking N-methyl D-aspartate (NMDA) receptors [103], nicotinic receptors and 5-HT3 receptors: the results showed a decreased RWA, increased food intake, lower corticosteroids levels and the absence of amenorrhea in agmatine-treated ABA rats [104].

#### γ-Aminobutyric acid (GABA)

A dysregulation of the GABA system is often associated with anxiety symptoms, usually present in human patients with AN [105, 106]. For example, facilitation of GABA_A_ receptors signaling has proven to restore feeding behavior in adult mice where hypothalamic neurons expressing the neuropeptide Y (NPY) and the agouti-related protein AgRP were ablated leading to reduced eating [107]. The modulation of the hypothalamic GABAergic signaling, through subcutaneous administration of kisspeptin (a neuropeptide controlling the reproductive function of the body), also demonstrated to reinforce food consumption in ABA rats [108]. The α4 subunit of GABA receptors (α4βδ-GABARs) is known to be regulated by gonad hormone fluctuations [109]. In adolescent ABA rats, α4βδ-GABARs expression seemed to be increased in proximity of GABAergic interneurons within the amygdala circuits, thus compromising inhibition. This resulted in an augmented excitation, leading to increased anxiety-like behaviors [110]. Conversely, resilience to the ABA protocol (i.e. suppression of the running wheel hyperactivity evoked by the protocol) was positively related to the up-regulation of α4βδ-GABAA receptors at pyramidal CA1 neurons of dorsal hippocampus, thus contributing to anxiolysis of stressed animals and to suppression of excessive exercise [110, 111]. The hippocampal insertion of α4βδ-GABARs is also known to be up-regulated by allopregnanolone (3α-OH-5α-pregnan-20-one; THP), whose administration reduces anxiety [112, 113]. Another work demonstrated the upregulation of α4βδ-GABARs at dorsal hippocampal pyramidal cells in resilient female ABA mice [114]. Unexpectedly, when these mice were treated with progesterone, which is rapidly converted into its metabolite THP, and subsequently subjected to a second ABA protocol, RWA was promoted; this inverse effect was attributed to THP ability to desensitize α4-GABAARs in the dorsal hippocampus, unmasking many glutamatergic receptors, consequently making neurons hyperexcitable [115]. Therefore, concerns arose about the supposed efficiency of estrogens in treating AN, as well as those of benzodiazepines, often ineffective in reducing anxiety shown by patients with AN [111]. GABA transmission in mPFC might also be affected in ABA mice through the morphological changes in GAD + terminals length reported above [116].

#### Cannabinoids

According to a study conducted in obese rats, CB1R (cannabinoid type-1 receptor, densely expressed at hypothalamus and basal ganglia) and OBR (leptin receptor) seem to operate in synergy by modulating neuroendocrine and behavioral ABA responses [117]. A second study found an increased binding of [18F]MK-9470, a selective high-affinity inverse agonist for CB1R, in all cortical and subcortical regions of ABA rats; hippocampus, bilateral inferior colliculus and entorhinal cortex were also involved, but only in female ABA animals [118]. Alteration of the endocannabinoid system in the brain of ABA rats was also evidenced thanks to the analysis of CB1R density, found to be decreased in the dentate gyrus of hippocampus and in the lateral hypothalamus; after ABA induction, lower levels of different endocannabinoids and some related lipids were also found in different brain areas, with a partial normalization after recovery [119]. CB1R gene was also found downregulated in the hypothalamus and NAc of ABA rats, together with an increased promoter methylation in the second area [120]. In addition, there is evidence that tetrahydrocannabinol (THC), if administered to ABA rats, increases their food intake, despite not acting on survival [121]. Similarly, using THC receptor agonists, female rats subjected to ABA protocol showed a reduction of body weight loss as well as a transient stimulation of food intake and a moderate reduction of the RWA. Moreover, the expression of genes associated with brown adipose tissue thermogenesis and white adipose tissue lipid metabolism were consistent with a reduction in energy expenditure and lipolysis, known to increase when THC treatment is associated with a high fat diet [122].

#### Opioids

Opioid system genetic variations seem to be involved in AN [90, 123] and in the development of addiction leading to excessive physical activity [124, 125]. Knockout mice for the opioid-μ receptor, subjected to ABA protocol, showed a reduced level of FAA, interpreted as a result of a change in the activation of the dopamine system [126]. Indeed, it is known that reward anticipation behaviors depend on the activity of mesolimbic dopaminergic pathways, regulated by opioid-μ receptors, probably as a response to the overcoming stress to achieve the goal [127].

### Brain-derived neurotrophic factor (BDNF)

BDNF is a neurotrophic factor actively involved in the expression of neuronal plasticity [128], including a promoting action on GABAergic synaptogenesis [116]. Since polymorphisms in the BDNF gene have been associated to higher probability of developing eating disorders [129], the role of BDNF in vulnerability to ABA has attracted interest. Adolescent female mice with the BDNF-Val66Met gene variant, which are characterized by deficient BDNF expression and increased anxiety-like behaviors, also showed an augmented probability of developing abnormal feeding behavior when subjected to caloric restriction and social isolation [130]. On the contrary, male adolescent mice with the same BDNF-Val66Met variant showed less vulnerability to the ABA protocol, as well as reduced GABAergic innervation of pyramidal neurons in PFC and hippocampal CA1 [116]. Another study estimated the expression of BDNF at the mesocorticolimbic circuit [131]: food restriction alone increased BDNF transcription in hippocampus while reducing it in the mPFC, while RWA increased BDNF transcripts levels in VTA. Interestingly, the authors also found that, when both caloric restriction and RWA were combined, no changes in BDNF transcription were detected [131]. Nevertheless, it is worth noting that BDNF mRNA levels do not always correlate with protein levels [132].

### Neurohormones

Neurohormonal mediators and the hypothalamic–pituitary–adrenal (HPA) axis are strongly implicated in AN [133, 134] and the role of these hormones in the induction, maintenance and rescuing of the ABA phenotype are currently being investigated.

#### Leptin and ghrelin

Leptin is a hormone produced by adipose cells. It regulates the sense of satiety and the feeding behavior by activating hypothalamic circuits and by regulating thermogenesis, and its lack is related to pathological conditions such as obesity and hyperphagia [135]. Leptin is also known to induce fatty acids oxidation and glucose uptake [136]. Leptin levels provide information about the organism’s energetic state to the hypothalamic arcuate nucleus by binding to its receptors (LEPRs) and producing opposite actions on two different neural populations: cells co-expressing AgRP, NPY and GABA are inhibited, resulting in an orexigenic action; a second neural population, expressing pro-opiomelanocortin (POMC) and cocaine- and amphetamine-regulated transcript (CART), is activated and codes for the anorexigenic α-melanocyte-stimulating hormone (α-MSH) [137]. AN patients, because of their lowering body adiposity, are in a hypoleptinic state [138, 139] and this could act as a trigger for physical hyperactivity. In fact, only mouse strains with higher levels of anxiety (e.g. DBA), when exposed to the ABA protocol, increased RWA showing the greatest decrease in the plasmatic levels of leptin [140]. Another study by Exner and colleagues showed that rodents subjected to food restriction exhibited semi-starvation induced hyperactivity (SIH) and lower leptin levels; leptin administration blocked SIH even when already induced [138]. A decrease in RWA was also seen through direct injection of leptin into VTA [141]. Based on this result, the authors suggest that the increased RWA might be produced by a decrease of leptin signaling in the mesolimbic pathway. However, a recent study by Fraga and colleagues [142] did not replicate the aforementioned findings. Indeed, several studies showed how leptin can act on dopamine neurons in the midbrain reward system, especially those projecting to the NAc [143, 144], thus mediating the incentive effect of foods [145–147]. Hence, the effect of leptin injections in the VTA was likely to reduce dopaminergic drive at the NAc, thus reducing food intake and RWA. Given the inhibition of feeding, the use of this hormone as AN therapy is arguable and debated [148, 149].

Together with hypoleptinemia, patients with AN also show an increase of ghrelin [30, 150]. Ghrelin, produced by P/D1 gastric cells, acts by stimulating the appetite and is widely studied in relation to AN since it modulates the mesolimbic reward system by activating the dopaminergic pathway [151]. This hormone also increases physical exercise [152]. A work on ABA mice showed that ghrelin injections decreased FAA with no effects on weight loss, and increased RWA in the absence of food [153]. Ghrelin signaling suppression (through ghrelin receptor GHS-R1A knockout and ghrelin receptor antagonist administration) inhibited the food anticipatory activity in ABA models without altering their food intake and total RWA, paving the way for a potential therapeutic target to treat hyperactivity in AN patients [154]. The dual mechanism of ghrelin, stimulating both appetite and motor activity, could involve different neural pathways such as hypothalamic circuits of appetite and, as already mentioned, dopaminergic circuits [155]. After a day of ABA protocol, increased ghrelin and decreased leptin levels were found in visceral fat and gonads of animals, compared to other adipose tissues. In muscle, the active and inactive forms of the leptin receptor show a tissue-specific pattern, dependent on the metabolic fiber type: a predominance of the long active form of leptin receptor (LEPR) was found in oxidative soleus muscle of ABA rats, probably more prone to be regulated by leptin, a possible defensive mechanism to maintain energy homeostasis in those situations where the caloric intake and energy expenditure are unbalanced [156].

The gut microbiota of the ABA model was also investigated [157], pointing out a positive correlation between serum leptin levels and quantity of *Bifidobacterium* and *Lactobacillus*, and a negative correlation between the same hormone and the number of *Clostridium, Bacteroides* and *Prevotella*. Ghrelin correlation was the opposite of that found for leptin [158]. In ABA mice such gut microbiota alterations were found to be caused by food restriction and were not modified by physical activity; hypothalamic NPY and POMC mRNA levels, body weight, food intake and lean/fat masses were also found to be correlated to different bacterial units [159]. Moreover, a premature state of intestinal inflammation was found in ABA mice, displaying an increase of hypothalamic receptors for inflammatory cytokines and for plasma corticosterone. Toll-like receptor 4 (TLR4) resulted upregulated too, both in colon epithelial cells and in the intestinal macrophages, causing an increase of mucosal cytokines production; since TLR4 knock-out mice showed a higher susceptibility to the ABA protocol, the immune response in the present model need to be further investigated [160].

#### Melanocortins

The melanocortin system plays a key role in different endocrine and homeostatic processes and is involved in the regulation of energy balance and in the development of obesity [161]. A study showed that ABA rats, before entering the severe starvation phase, displayed increased mRNA levels of AgRP, NPY and melanin-concentrating hormone (MCH) in the arcuate nucleus, and decreased levels of POMC and CART in the lateral hypothalamus [162, 163]. As expected, stimulating the melanocortin system though a chronic treatment with α-MSH, known to be released from POMC neurons to decrease appetite and increase energy expenditure, worsened ABA symptoms [164–166]. Unexpectedly, POMC initial increase was lowered along the ABA protocol and central injections of SHU9119, a melanocortin MC3-4 receptors antagonist, did not result in RWA nor food intake decrease. The transient POMC increase could involve the opioid system: the endogenous release of β-endorphins by the hypothalamus, triggered by the ABA protocol, appears to be primarily involved in the motivation to acquire food reinforcements [167, 168].

Drug therapies designed to antagonize MC receptors could be combined with a temperature adjustment. It is well known that a continuous warming is beneficial to ABA animals [169] and that rising temperature from 21 to 32 °C can block ABA induction and reduces its related behavior by increasing food intake and reducing RWA [170–172]. In this context, a parallelism between the ABA model and AN patients should be evidenced: in fact, warming at 32 °C also showed a fivefold reduction in corticosterone levels in ABA rats if compared to a 21 °C housing [173], and this anxiolytic effect was also present in AN patients when exposed to a heated environment [174]. Furthermore, a lower hypothalamic expression of MC4 receptor, AgRP and POMC was quantified, representing an adaptive response aimed at stabilizing the energy balance in a raised temperature context [175]. Since the activation of NPY/AgRP-expressing neurons is known to promote food intake, orexigenic action of AgRP has been investigated in relation to C1q/TNF-related protein 13 (CTRP13), a hormone secreted by adipose tissue implicated in peripheral regulation of glucose metabolism [176]. Both CTRP13 and AgRP resulted upregulated in the hypothalamus of ABA mice and the two molecules seemed to regulate each other: AgRP administration produced an increase of CTRP13, while CTRP13 inhibited AgRP and suppressed food intake, reducing body weight.

#### The hypothalamic–pituitary–adrenal (HPA) axis

How stress affects sensitivity to the ABA protocol has also been also investigated [177, 178]. ABA is known to activate HPA and to release corticotropin-releasing hormone (CRH) [162]. The activation of corticotropin-releasing factor (CRF) neurons was also reported in ABA rats [179]. Although the wheel activity itself seems to lower anxiety in rats previously exposed to stressful conditions, BDNF levels increased in the striatum and anxiety, measured by the open field test, turned out to be positively correlated with RWA in the food restriction phase of the ABA protocol [115, 180]. Another study aimed at investigating whether ABA exposure would cause sustained changes induced by HPA axis activation [62]. Adolescent female rats, subjected to ABA protocol and tested in adulthood, showed more anxiety during tests, higher levels of plasma corticosterone and elevated CRH mRNA levels in the hypothalamic paraventricular nucleus and in the central nucleus of the amygdala, with long-lasting effects on anxiety. Comorbidity between the development of AN in adolescence and anxiety/depression was then investigated, deepening the role of ovarian hormones in adolescent rats subjected to ABA and their long-term effects on behavior and estrogen signaling [181]. The authors found that two subsequent protocol exposures during the middle or late adolescence were necessary to produce anxiogenic long-term effects and HPA axis activation, recording a reduction in estrogen receptor (ERβ) in the amygdala. An oophorectomy was performed in animals before puberty and this resulted in a lower RWA and food intake stimulation during the ABA protocol, even though an increase in anxiety occurred when long-term tested. Thus, the hormonal decrease, usually found in ABA and here exacerbated, was hypothesized to guide the anxious symptomatology.

## Discussion

Despite the ABA model has been very valuable as a tool for investigating the neurobiological correlates of spontaneous caloric restriction in a laboratory setting, clearly there exist some important limitations to its validity. At first, it cannot be able to replicate the complex cognitive and behavioral features of the human AN patient. Furthermore, the relevance of excessive physical activity in the development of the ABA phenotype, while on the one hand increases its face validity, on the other might hinder its significance for modeling different AN clinical profiles. Another central issue relates to the role of weight loss: indeed, this might account at least in part for the neurobiological changes observed, without a real implication of the behavioral phenotype. To exclude such hypothesis, the use of weight-paired controls (control animals whose feeding is dynamically regulated to match the same weight loss of ABA rats) is always desirable. Unfortunately, such approach is very complex and thus quite uncommon.

A further limitation is that, despite their commonalities, all the ABA studies reviewed here present slight differences in the induction protocol, which therefore never matched perfectly. Food access was blocked for most of the day but during a short time window: besides its exact timing during the day or night, the overall length of food access varied from 60 min to 3 h [182]. As a further example, while the animals were allowed to eat, the wheel rotation could be either blocked [183] or still active; in the latter case the animal had to choose between eating or continuing the physical exercise. Thus, this heterogeneity from one study to another might explain the subtle differences found in results. For example, food intake should be greater when food is accessible and the exercise wheel blocked, compared to those protocols where the wheel is still working and accessible [37, 40, 71, 87]. The consideration that motivation for running on the wheel, physical activity and performance are autonomous entities helps understanding the different reward processes underlying the induction of AN in the ABA model [184], alongside designing ad hoc neurocognitive assays to clarify the role of cognition in AN [185]. The exact timing of food access relates to the fact that rodents typically eat more and have greater physical activity during the dark phase. In some studies, the two phases of the light–dark cycle were inverted, thus supplying food during daily hours. Developing a consensus for the most effective AN induction protocol and eliminating potential sources of variabilities among studies would be desirable. In addition, restricting the analysis to specific predictors, either of the ABA phenotype or of the genetic of rodent lines, may help clarifying the vulnerability determinants to develop AN using the ABA model [19, 88, 186, 187]. Testing different strains would then be necessary to map a wide range of quantitative trait loci with the aim to investigate the molecular determinants in the onset and development of AN [188]. Also, testing resilient models would help classifying resistance genes and neural substrates that might be useful when developing more effective therapeutic strategies [183]. Although the aforementioned limitations, the ABA model will continue providing novel results and insights on AN in the coming years, also in light of the possibility to integrate it with recent technical advances that allow refined measurements of neural and synaptic activity and its manipulation [189–191], as well as with the use of specific transgenic models to conduct a morphological analysis on the ABA phenotype of wild type and diseased synapses, e.g. in specific motor pathways [192].

As mentioned in this work and previously discussed by other authors [87], the most likely hypothesis for the mechanism behind the development of the ABA phenotype relates to an imbalance of the neural circuitry that mediates reward and hedonic value processing, where RWA replaces food as the predominant reinforcement in an operant conditioning perspective. This is supported by the accumulating evidence about the implication of the ventral mesolimbic reward pathway as well as the action of dopaminergic antagonists in modulating hyperactivity and feeding in ABA rodents. In the near future it would be important to further explore and dissect out these mechanisms, including those elements which have not been addressed yet, such as the role of receptors and molecules controlling or modulating synaptic plasticity and the related metaplastic phenomena.

Nevertheless, the evidence collected in this review suggests that ABA animals show a large set of alterations, both physiological and anatomical, involving regions and circuits which are not directly implicated in the reward processing and, based on what is known today, whose functions extend way beyond the control of reward mechanisms and eating habits. Hence, we cannot exclude a primary role of these alterations from a mechanistic theory of ABA induction. This consideration is far from representing a limitation to the validity of the model. On the contrary, higher order cognitive functions, such as explicit memory, decision making, cognitive flexibility and attention, are known to be altered in AN and are being recently found modified in ABA rodents [34]. More importantly, many AN triggering factors are related to cognition, such as emotional distress, body image distortion and obsessive–compulsive traits. In this prospect, although the reward system is clearly implicated in the development and maintenance of the ABA condition, pre-existing individual differences in cognitive functioning might implicate vulnerability of the animal [183]. We believe that such view might increase construct validity of the ABA model thus deserving consideration in the design of future studies.

## Conclusions

The findings collected here are not sufficient to clarify the neurobiological mechanisms behind the development of AN, still they can drive the design of novel ad hoc investigations. The prospect is that the ABA model could be more effectively used to shed light on the complex AN neurobiological framework, thus supporting the development of more effective therapeutic strategies.

## Data Availability

Not applicable.

## Abbreviations

5-HT: Serotonin
ABA: Activity-based anorexia
AgRP: Agouti-related protein
AMPA: α-Amino-3-hydroxy-5-methyl-4-isoxazolepropionic acid
AN: Anorexia nervosa
BDNF: Brain-derived neurotrophic factor
CART: Cocaine- and amphetamine-regulated transcript
CB1R: Cannabinoid type-1 receptor
CRF: Corticotropin-releasing factor
CRH: Corticotropin-releasing hormone
CTRP13: C1q/TNF-related protein 13
DA: Dopamine
DSM-5: Diagnostic and statistical manual of mental disorders, 5th edition
ERβ: Estrogen receptor
FAA: Food anticipatory activity
GABA: γ-Aminobutyric acid
GABAR: γ-Aminobutyric acid receptors
HPA: Hypothalamic–pituitary–adrenal
LEPRs: Leptin receptors
MDMA: 3,4-Methylenedyoximethamphetamine
MHC: Melanin-concentrating hormone
mPFC: Medial prefrontal cortex
NAc: Nucleus accumbens
NMDA: N-methyl D-aspartate
NPY: Neuropeptide Y (NPY)
PFC: Prefrontal cortex
POMC: Pro-opiomelanocortin
RWA: Running wheel activity
SIH: Starvation induced hyperactivity
THC: Tetrahydrocannabinol
TLR4: Toll-like receptor 4
VTA: Ventral tegmental area
α-MSH: α-Melanocyte-stimulating hormone
